# High-throughput bioengineering of homogenous and functional human-induced pluripotent stem cells-derived liver organoids *via* micropatterning technique

**DOI:** 10.3389/fbioe.2022.937595

**Published:** 2022-08-10

**Authors:** Xiaodong Xu, Shanqing Jiang, Longjun Gu, Bin Li, Fang Xu, Changyong Li, Pu Chen

**Affiliations:** ^1^ Tissue Engineering and Organ Manufacturing (TEOM) Lab, Department of Biomedical Engineering, Wuhan University TaiKang Medical School (School of Basic Medical Sciences), Wuhan, China; ^2^ Hubei Province Key Laboratory of Allergy and Immunology, Wuhan University TaiKang Medical School (School of Basic Medical Sciences), Wuhan, China; ^3^ Institute of Hepatobiliary Diseases of Wuhan University, Hubei Engineering Center of Natural Polymers-based Medical Materials, Zhongnan Hospital of Wuhan University, Wuhan, China

**Keywords:** liver organoids, human induced pluripotent stem cells, micropatterning technique, homogeneity, hepatotoxicity

## Abstract

Human pluripotent stem cell-derived liver organoids are emerging as more human-relevant *in vitro* models for studying liver diseases and hepatotoxicity than traditional hepatocyte cultures and animal models. The generation of liver organoids is based on the Matrigel dome method. However, the organoids constructed by this method display significant heterogeneity in their morphology, size, and maturity. Additionally, the formed organoid is randomly encapsulated in the Matrigel dome, which is not convenient for *in situ* staining and imaging. Here, we demonstrate an approach to generate a novel type of liver organoids via micropatterning technique. This approach enables the reproducible and high-throughput formation of bioengineered fetal liver organoids with uniform morphology and deterministic size and location in a multiwell plate. The liver organoids constructed by this technique closely recapitulate some critical features of human liver development at the fetal stage, including fetal liver-specific gene and protein expression, glycogen storage, lipid accumulation, and protein secretion. Additionally, the organoids allow whole-mount *in-situ* staining and imaging. Overall, this new type of liver organoids is compatible with the pharmaceutical industry’s widely-used preclinical drug discovery tools and will facilitate liver drug screening and hepatotoxic assessment.

## 1 Introduction

As an essential metabolic organ of the human body, the liver performs a variety of fundamental physiological functions, including detoxification, digestion, and metabolism. Liver dysfunction may arise in many hepatic diseases and can also be caused by various factors such as viruses, alcohol use, and drug-induced liver injury (DILI). Among these factors, DILI is a major cause of acute liver failure (ALF) and one of the leading indications for liver transplantation (Chalasani et al., 2015; Xu et al., 2022). It's reported that the annual incidence of DILI in the general population ranges between 13 and 24 cases per 100,000 inhabitants (Sgro et al., 2002; Bjornsson et al., 2013; Shen et al., 2019; Kamath et al., 2021). DILI is a rare potential complication in pregnant women, but it can adversely affect both the mother and the fetus. Clinical trials seldomly study drug’s hepatoxic effects in pregnant women due to ethical and safety concerns unless the drug is specifically used in pregnant women (Kamath et al., 2021). It is therefore essential to establish a research model relevant to human fetal livers in order to study the drug’s hepatotoxicity in fetuses.

Pluripotent stem cell (PSC) derived human liver organoids (HLOs) are emerging humanized liver models and are have been increasingly used in mechanistic studies of liver diseases and evaluation of drug’s hepatotoxicity. Compared with the two-dimensional (2D) hepatic cell culture model, PSC-derived HLOs can reconstitute three-dimensional (3D) intercellular and heterocellular communications as well as cell-extracellular matrix interactions in the native liver microenvironment and thus better emulate liver physiological functions and tissue homeostasis (Ouchi et al., 2019). Moreover, unlike liver tissue-derived HLOs that are specified to the endoderm fate and only contain hepatic parenchymal cells, PSC-derived HLOs can contain both parenchymal and nonparenchymal cells of liver tissue (Goulart et al., 2019; Velazquez et al., 2021). Furthermore, PSC-derived HLOs are humanized and have the developmental trajectory and metabolic characteristics more relevant to human liver than animal models.

At present, the Matrigel dome method is a gold standard for generating PSC-derived HLOs. Specifically, PSCs or PSC-derived stem cells are encapsulated in the Matrigel dome and experience multistage differentiation and culture, and ultimately form hundreds of heterogeneous liver organoids in the Matrigel dome (Ouchi et al., 2019; Shinozawa et al., 2021). However, the initial cell encapsulation process is highly randomized. A complex cell microenvironment, especially nonuniform inter-organoid communications, leads to great heterogeneity in organoid formation within or between individual domes, manifested explicitly in morphology, size, and maturity. Moreover, randomly distributed organoids in the dome are not convenient with whole-mount *in-situ* imaging and long-term the same single organoid monitoring. These issues seriously hamper PSC-derived HLOs’ wide application in liver diseases and hepatotoxicity.

In this study, we demonstrate a simple and reliable method to generate deterministic liver organoids from human-induced pluripotent stem cells (hiPSCs). We employed the hiPSC micropatterning technique to define adhesion regions of hiPSCs and therefore determine the location, arrangement, and size of individual organoids at the bottom of a standard multiwell plate. To generate hiPSC-derived HLOs on the micropatterned cell-adhesion substrate (MPCS), we initially differentiated hiPSCs into foregut stem cells (FSCs). We then seeded FSCs on the MPCS for subsequent liver differentiation. During the differentiation, morphogenesis and functional maturation of individual organoids are regulated due to the constrained physical boundary of organoid growth and deterministic inter-organoid locations. Therefore, micropatterned HLOs (mpHLOs) were homogeneous in size, morphology, and maturity. Furthermore, we characterized the mpHLOs by liver-specific biomarkers and functional assays. Finally, we used the mpHLOs as a human-relevant fetus liver model to study the fetal hepatotoxicity of drugs.

## 2 Materials and methods

### 2.1 Fabrication of micropatterned cell-adhesion substrate

**FIGURE 1:**
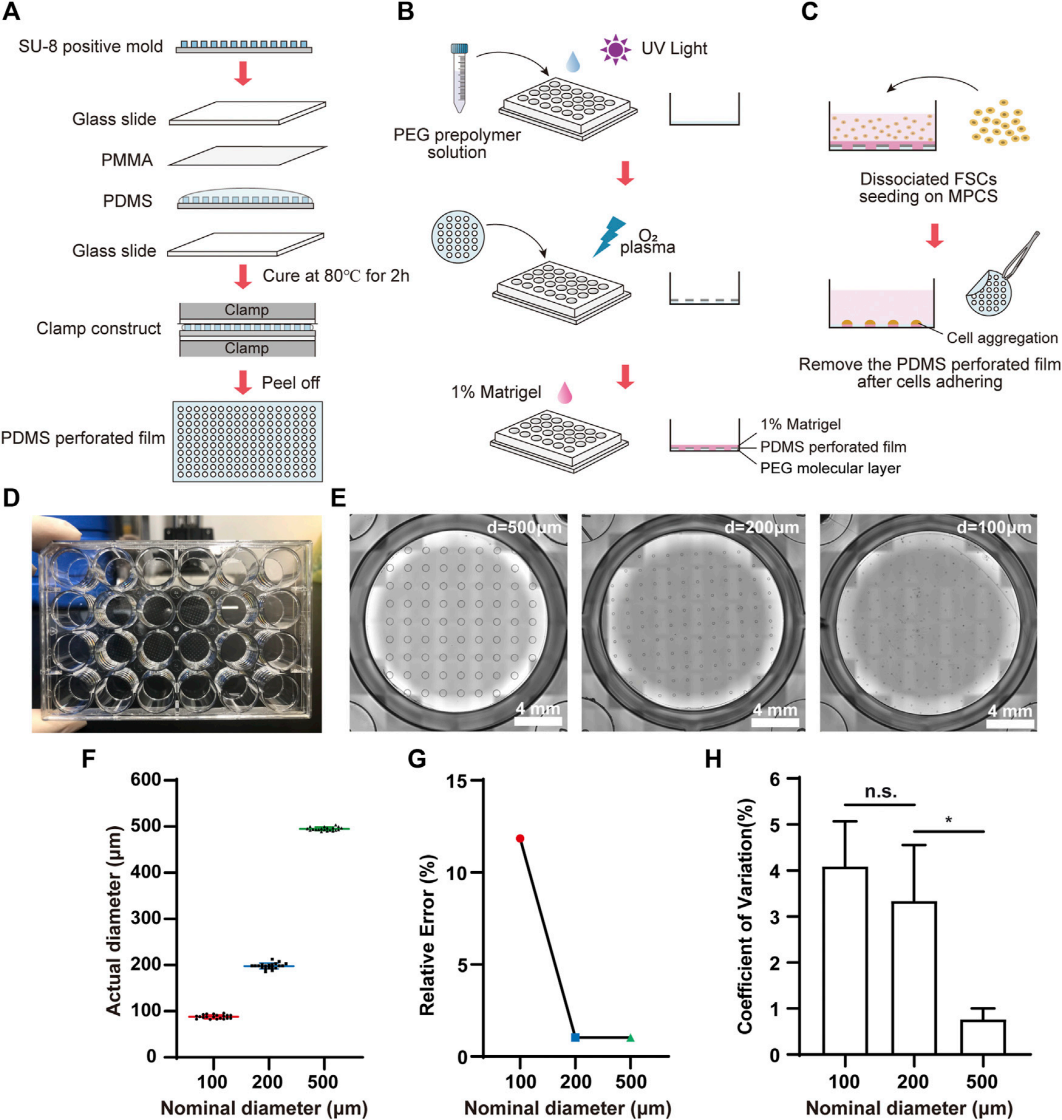
Fabrication and characterization of MPCS. Schematic diagram of **(A)** the fabrication process for perforated PDMS film, **(B)** PEG coating, and **(C)** cell seeding on the MPCS. **(D)** Photograph of MPCS. **(E)** Bright field images of MPCS with circular patterns of 500 μm, 200 μm, and 100 μm in diameter from left to right, respectively. **(F)** Scatter plot (mean ± SD, *n* = 20), **(G)** Relative error (*n* = 20), and **(H)** CVs of actual diameters of the perforated PDMS film (*n* = 6). Data are the mean ± SD and analyzed by One-way analysis of variance, *p < 0.05, **p < 0.01, ***p < 0.001, ****p < 0.0001.

We fabricated MPCS for the organoid formation and culture (Figures 1A,B). Briefly, the MPCS was fabricated in a 24-well plate by oxygen plasma treatment of a perforated-PDMS-film-covered PEG surface. The perforated PDMS film was fabricated by clamping and solidifying PDMS prepolymer on a SU-8 positive mold (Figure 1A). The SU-8 positive mold was fabricated using the standard photolithography method with a SU-8 photoresist (Ma et al., 2015). Component A and component B of PDMS prepolymer were mixed with a ratio of 10:1 and degassed via a vacuum pump. The degassed PDMS prepolymer was pipetted onto a SU-8 positive mold and then covered by a 0.2 mm thick PMMA slide. Next, the PMMA-PDMS-SU-8 complex was sandwiched by two glass slides and a bench clamp. The PDMS prepolymer in the complex was cured in an 80°C oven for 2 hours. The plate was first treated with oxygen plasma at 550 W for 1 min and then covered with PEG prepolymer solution (Figure 1B). The PEG prepolymer solution in a 50-ml tube is comprised of 0.15 g of PEG 1000, 1.8 ml of PEG 400, 14.55 ml of Isopropyl alcohol (IPA), and 0.45 ml of Milli-Q water. The prepolymer solution was vortexed for at least 3 min for homogenization. A 40 mg of Irgacure 2,959 was then dissolved into the prepolymer solution. Then, the prepolymer solution was added to a 24-well plate and photo-crosslinked with ultraviolet exposure for 1 min using a photolithography machine. The plate was washed with 70% alcohol three times to remove the remaining PEG monomers (Hoang et al., 2018). The PEG-coated plate was covered by the tailored perforated PDMS film and placed at 80°C ovens for 10 min. Then the plate was treated three times with 4-min oxygen plasma at 700 W with an interval of 3 min to remove the PEG regions not covered by the PDMS film. Before cell seeding, the PEG-coated plate was sterilized by UV for 1 hour, washed with Dulbecco’s phosphate-buffered saline (DPBS), and coated with 1% (v/v) Matrigel for 1 hour in an incubator. DPBS was obtained by ten-fold dilution of (10×DPBS) with Milli-Q water. All reagent information in this article is given in Supplementary Table S1.

### 2.2 Human-induced pluripotent stem cells maintenance culture

HiPSCs were maintained in Nuwacell^TM^ ncTarget hPSC Medium (ncTarget), passaged via Accutase detachment, and reseeded in the Vitronectin (VTN)-coated 6-well plate containing ncTarget with 10 μM Y-27632.

### 2.3 Foregut induction

HiPSCs were differentiated into FSCs using a previously reported method with minor modifications (Thompson and Takebe, 2020). Briefly, hiPSCs were detached by Accutase and were seeded on VTN-coated tissue culture plates with 100,000 cells/cm^2^. Differentiation was started when the cell confluence reached 85–90%. The medium was changed to RPMI 1640 medium containing 100 ng/ml Activin A and 50 ng/ml bone morphogenetic protein 4 (BMP4) on Day 1, 100 ng/ml Activin A and 0.2% Knockout serum replacement (KSR) on Day 2, and 100 ng/ml Activin A and 2% KSR on Day 3. On Day 4–6, the cells were cultured in Advanced DMEM/F12 with B27 and N2 containing 500 ng/ml fibroblast growth factor-2 (FGF2) and 3 μM CHIR99021. Cells were maintained at 37°C in 5% CO_2_ with 95% air, and the culture medium was changed every day.

### 2.4 Micropatterning of human-induced pluripotent stem cells-derived foregut stem cells

On Day 6, FSCs were detached by Accutase for 3 min at 37°C, then suspended in Advanced DMEM/F12 with B27 and N2, containing 80 ng/ml FGF2, 3 μM CHIR99021, and 10 μM Y-27632. The FSCs were seeded on the MPCS with a seeding concentration of 3.2×10^5^ cells/cm^2^. At 4-h after seeding, the FSCs adhered to the substrate, and the PDMS film was then carefully removed from the substrate using a sharp tweezer, leaving the micropatterned FSC regions (Figure 1C). Then the FSCs were washed with DPBS gently. The medium was replaced by the same formula above without the addition of Y-27632. The cells were maintained at 37°C in 5% CO_2_ with 95% air.

### 2.5 Generation of human liver organoids

On Day 6, the FSCs were seeded on the MPCS and cultured in Liver Organoid Formation Medium for 4 days. The medium was replaced every 2 days. On Day 10, the culture medium was switched to Liver Organoid Specification Medium and replaced every 2 days until Day 14. Then, the culture medium was switched to a Complete hepatocyte culture Medium and replaced every 2 days until Day 24. HLO induction and culture were maintained at 37°C in 5% CO_2_ with 95% air.

### 2.6 RNA-seq and data analysis

On Day 24, mpHLOs were washed gently three times with DPBS. The total RNAs of mpHLOs were extracted following the Total RNA Extraction Reagent (Trizol) manually. The library and sequencing of transcriptome were prepared using Illumina HiSeq X Ten (Novogene Bioinformatics Technology Co., Ltd., Beijing, China). The mapping of 100-bp paired-end reads to genes was undertaken using HTSeq v0.6.0 software, while fragments per kilobase of transcript per million fragments mapped (FPKM) were also analyzed.

### 2.7 Real-time quantitative PCR

Total mRNAs were isolated from the cells and HLOs using the Trizol reagent. cDNAs were synthesized using ABScript Ⅲ RT Master Mix. qPCR was performed using 2X Universal SYBR Green Fast qPCR Mix denaturation at 95°C for 1 min, annealing at 58°C for 30 s, and extension at 72°C for 30 s. All primers' information is described in Supplementary Table S1. The expression levels were normalized relative to the expression of the housekeeping gene GAPDH using the comparative Ct-method 2^-△△*Ct*^.

### 2.8 Immunochemistry and image analysis

For monolayer hiPSCs culture, the cells were fixed with 4% (w/v) paraformaldehyde for 15 min at room temperature (RT). Then the cells were blocked with 1% (w/v) bovine serum albumin (BSA) for 1 hour, incubated with primary antibodies overnight at 4°C, and washed three times with DPBS for 5 min. Then the cells were incubated with secondary antibodies for 2 hours at RT. After the reaction, the cells were washed three times with DPBS for 15 min.

For 2.5D organoid culture, the cells were fixed with 4% (w/v) paraformaldehyde overnight, permeabilized twice with 0.5% (v/v) Triton X-100 on a shaker for 20 min each time at a speed of 60 rpm, and blocked with 1% (w/v) BSA and 5% (v/v) Triton X-100 for 1 hour on a shaker at RT. Unless otherwise specified below, the rotation speed of the shaker was 60 rpm. Antibody diluent was prepared from DPBS, 1% (w/v) BSA and 0.1% (w/v) saponin. The samples were then incubated with primary antibodies for 24 h on a shaker at 4°C and washed three to four times with 0.5% (v/v) Triton X-100 for 20 min each time. Subsequently, secondary antibodies were incubated for at least 24 h on a shaker at 4°C. The samples were washed three times with 0.5% (v/v) Triton X-100 for 10–20 min each time. Finally, cell nuclei were stained with 4,6-diamidino-2-phenylindole (DAPI), and the fluorescence images were taken using an Olympus inverted microscope. ImageJ software analyzes the average fluorescence intensity of the images.

Detailed antibody information and dilution ratio is described in Supplementary Table S1 in the supplementary information.

### 2.9 Periodic-acid-Schiff and nile red staining assays

On Day 24, liver organoids were fixed in 4% (w/v) paraformaldehyde at 4°C overnight. The fixed organoids were then washed with DPBS and transferred to a 15% (w/v) sucrose solution at 4°C overnight, then to a 30% (w/v) sucrose solution for dehydration. HLOs were frozen and sectioned into 10 µm-thick slices using a cryostat. These cryosections of the HLOs were stained using a Periodic-acid-Schiff (PAS) reagent kit to visualize glycogen synthesis. The cryosections were stained with a 500 nM Nile Red reagent to visualize intracellular lipid accumulation. The stained cryosections were washed twice with DPBS to remove excessive reagents. Image acquisition was conducted either with a bright-field microscope or a confocal epi-fluorescence microscope.

### 2.10 Elisa analysis of albumin and urea detection assays

On Days 10, 14, and 24, the culture media over the past 48 h were collected from individual wells and stored at −80°C for albumin and urea measurement. Albumin concentration in the medium was measured using the Human Albumin ELISA Kit. Urea concentration in the medium was determined using the QuantiChrom Urea Assay Kit.

### 2.11 Statistical analysis

All data were analyzed using GraphPad Prism version 8.0.2 for Windows. Data were presented as mean ± SD. Shapiro-Wilk test was used for normal distribution assay. For single comparisons, a Student’s t-test was used. For multiple comparisons, one-way analysis of variance (ANOVA) was used with a Bonferroni post-test. Statistical significance of variables following skewed distribution was determined by Kruskal–Wallis test or Dunn’s multiple comparison test. ****p < 0.0001, ***p < 0.001, **p < 0.01, *p < 0.05, n. s. = not significance (*p* > 0.05). The sample sizes were indicated in the Figure legends.

## 3 Results

### 3.1 Characterization of micropatterned cell-adhesion substrate

We fabricated the MPCS (Figures 1A–D) and then characterized the micropatterns using bright-field image analysis. The MPCS was fabricated with a size-varied circular micropattern (500 μm, 200 μm, and 100 μm in diameter) (Figure 1E). The image analysis indicated that the micropatterning technique enabled fabricating circular micropatterns with an actual diameter consistent with the nominal diameter (Figure 1F). Specifically, the micropatterning technique allowed less than 2% relative error for fabricating micropatterns larger than 200 μm (Figure 1G). Additionally, the fabricated micropatterns were highly repeatable, with a coefficient of variation (CV) of less than 5% in their sizes (Figure 1H).

### 3.2 Characterization of human-induced pluripotent stem cells-derived micropatterned HLOs

**FIGURE 2:**
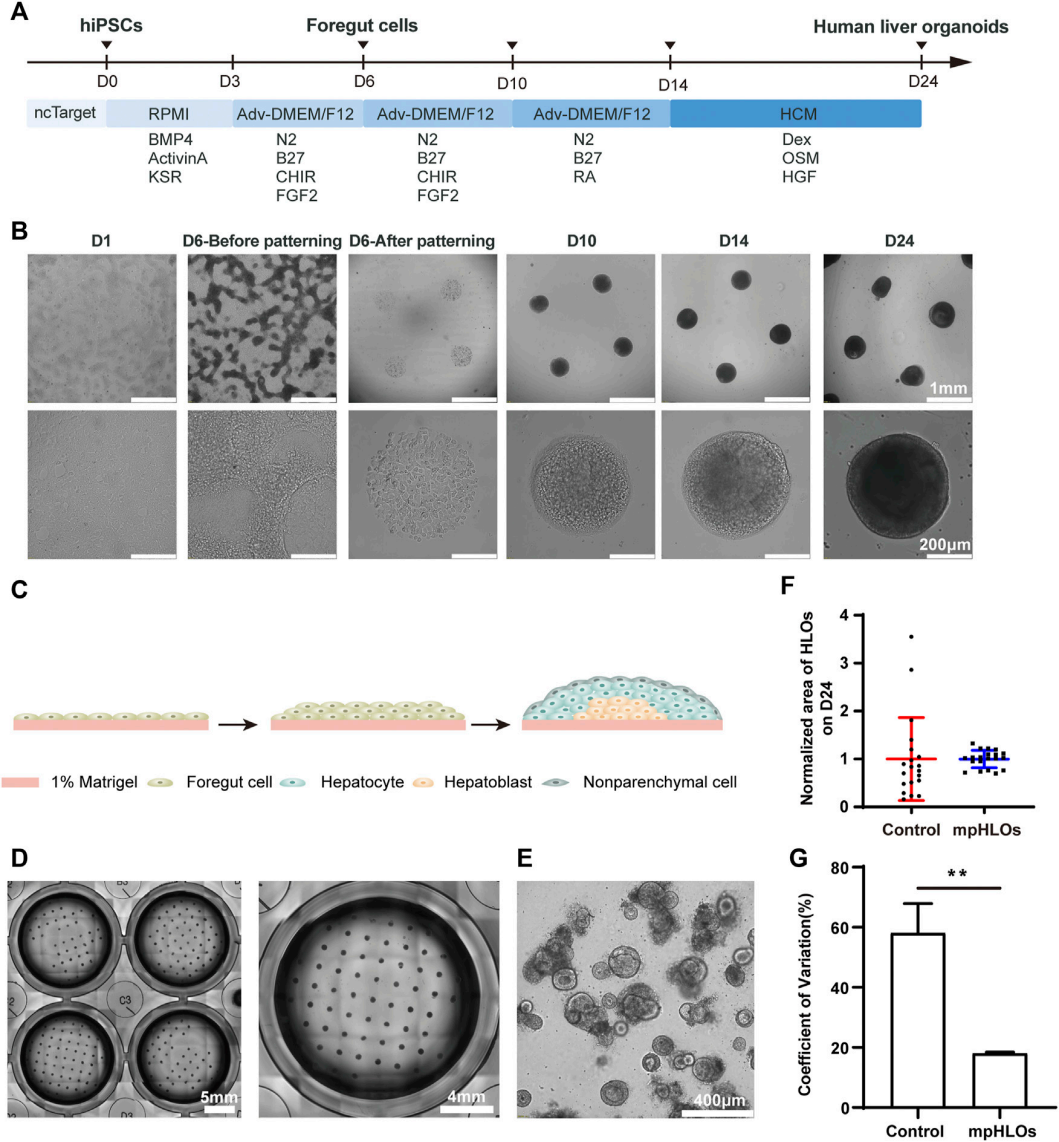
Characterization of hiPSC-derived mpHLOs. **(A)** Culture timeline diagram of mpHLOs. **(B)**Time-lapse images of generation of mpHLOs. mpHLOs are circular patterns with a diameter of 500 μm. **(C)** Schematic diagram of mpHLOs. **(D)** Bright-field images of mpHLOs in 24-well plate. **(E)** HLOs in Matrigel dome. **(F)** Normalized area of HLOs (*n* = 20). **(G)** CVs of HLOs (*n* = 6). Data are the mean ± SD and analyzed by Student’s t test, *p < 0.05, **p < 0.01, ***p < 0.001, ****p < 0.0001.

We generated mpHLOs via two-stage differentiation from hiPSCs (Figure 2A). Briefly, monolayer-cultured hiPSCs were first differentiated into FSCs using a previously reported method with minor modifications (Thompson and Takebe, 2020). On Day 6, FSCs formed, indicated by the spontaneous occurrence of stratification and cell aggregation (Figure 2B). FSCs were dissociated into single cells, then seeded on the MPCS for subsequent 18-day culture and differentiation. On Day 10, the micropatterned monolayer cells formed a dome-shaped multilayer cytoarchitecture. The cytoarchitecture became thick and opaque in the subsequent culture until Day 24 (Figures 2B,C).

The mpHLOs were compatible with the widely-used standard multiwell-plate culture system. A single well in the 24-well plate could accommodate a maximum 65 of 500 μm-sized HLOs with an inter-space of 1.5 mm (Figure 2D). Conversely, HLOs generated by the Matrigel dome method were randomly distributed in the Matrigel and showed significant heterogeneity in their size and morphology (Figure 2E). The bright-field image analysis indicated that the mpHLOs on Day 24 displayed a much lower area dispersion than the HLOs in the Matrigel dome method (Figure 2F). Specifically, the area CVs of mpHLOs are significantly lower than that of HLOs in Matrigel domes (Figure 2G).

### 3.3 Characterization of liver-specific gene and protein expressions

**FIGURE 3:**
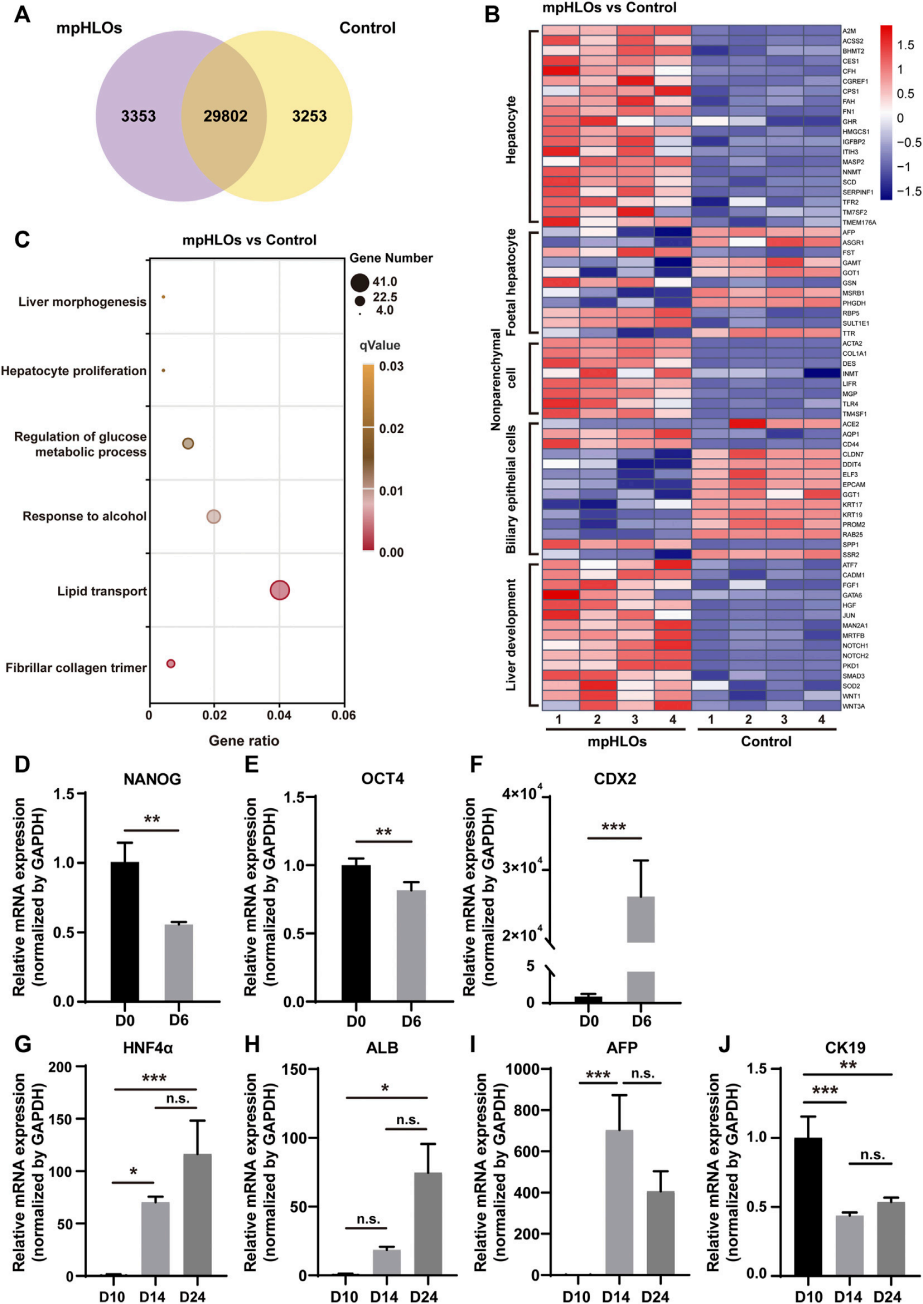
The transcriptomic analysis and relative mRNA expression of key genes in differentiation process. **(A)** Venn diagram shows the number of co-expressing and differentially expressing genes between mpHLOs and HLOs in the Matrigel dome (Control). **(B)** Heat map shows gene expression in hepatocyte, fetal hepatocyte, nonparenchymal cell, biliary epithelial cell, and liver development. **(C)** Bubble diagram indicates up-regulated genes enrichment by Gene Ontology analysis. **(D)** The pluripotency gene NANOG and **(E)** OCT4. **(F)**The posterior foregut gene CDX2. **(G)** The hepatic gene HNF4α. **(H)** The hepatic gene ALB. **(I)** The hepatic progenitor gene AFP. **(J)** The cholangiocyte gene CK19. Gene expression is shown as fold changes during two-week differentiation relative to the levels in D10 group, as determined by qPCR. Data are the mean ± SD (*n* = 3) and analyzed by Student’s t test or One-way analysis of variance, *p < 0.05, **p < 0.01, ***p < 0.001, ****p < 0.0001.

To further characterize the global gene expression profile of mpHLOs, we performed RNA sequencing of cells dissociated from mpHLOs on Day 24. Compared with the conventional Matrigel dome group, 29802 genes co-expressed in both the groups and 3,253 genes were uniquely expressed in the MPCS group (Figure 3A). We performed a differential gene expression (DGE) analysis of liver development and cell specification genes. The result indicated that mpHLOs showed higher expression of matured hepatocyte-specific genes and liver non-parenchymal cell-specific genes. The control group showed higher expression of biliary epithelial cell-specific genes and fetal hepatocyte-specific specific genes. Simultaneously, mpHLOs showed higher liver development-related gene expression than the control group (Figure 3B). Furthermore, gene ontology (GO) term enrichment analysis indicated that the biological processes enriched in this gene set were associated with liver functions such as lipid transport, response to alcohol, and regulation of glucose metabolic process (Figure 3C).

We characterized liver-specific gene and protein expressions in the mpHLOs using qPCR and immunofluorescence analysis. On Day 6, the mRNA expression levels of the pluripotency-associated stem cell markers, NANOG and OCT4, were decreased. In contrast, posterior foregut marker CDX2 significantly was increased (Figures 3D–F), revealing that hiPSCs were differentiated into FSCs. The mRNA levels of hepatic markers, albumin (ALB), and hepatocyte nuclear factor 4 alpha (HNF4α) were upregulated over 14-day differentiation since Day 10 (Figures 3G,H). Moreover, the mRNA expression of hepatoblasts marker α-fetoprotein (AFP) was increased from Day 10 to Day 14 and then decreased from Day 14 to Day 24 (Figure 3I). The above results indicated hepatic specification and maturation of the mpHLOs. In addition, the cholangiocyte marker cytokeratin 19 (CK19) was identified at distinct stages of organoid differentiation (Figure 3J), indicating the immature state of the mpHLOs.

**FIGURE 4:**
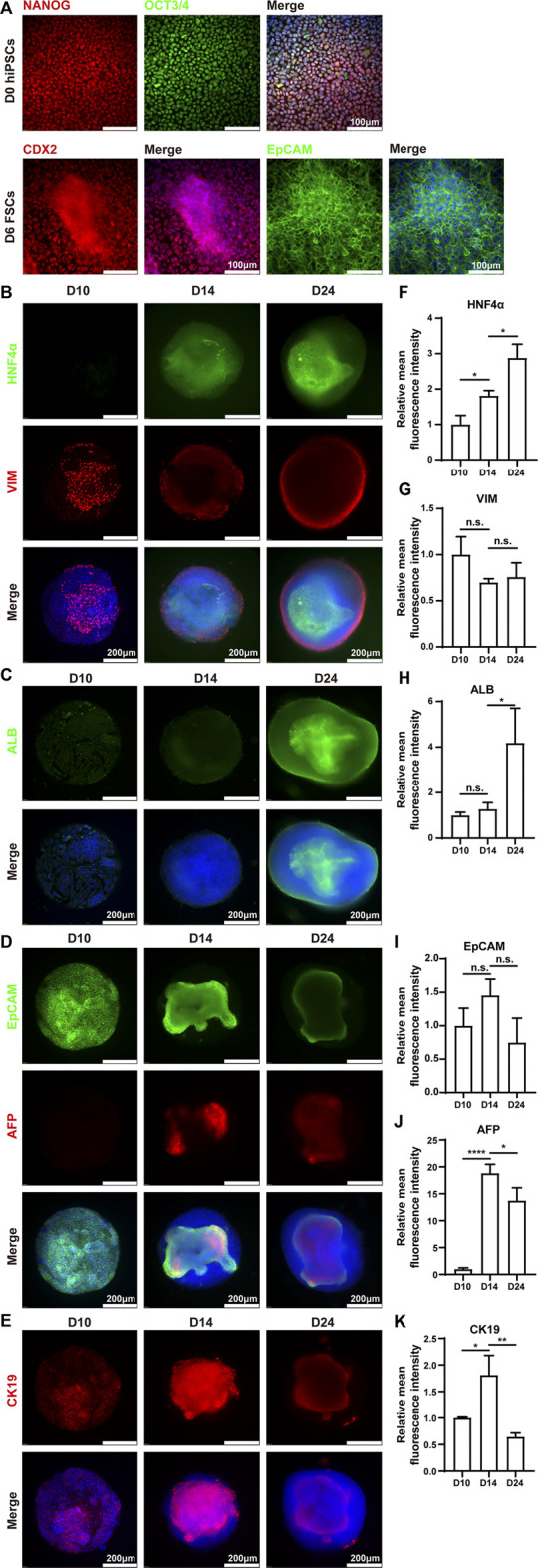
Immunohistochemistry analysis of mpHLOs. **(A)** Immunofluorescence staining of hiPSCs and FSCs for pluripotency markers NANOG and OCT4, posterior foregut marker CDX2, epithelial marker EpCAM. Immunofluorescence staining of mpHLOs for **(B)** hepatic maker HNF4α and mesenchymal marker VIM, **(C)** hepatic marker ALB, **(D)** epithelial marker EpCAM and hepatoblasts marker AFP, and **(E)** cholangiocyte marker CK19. **(F–K)** Semi-quantification analysis of immunofluorescence staining. Data are presented as the mean ± SD (*n* = 3) and analyzed by One-way analysis of variance, *p < 0.05, **p < 0.01, ***p < 0.001, ****p < 0.0001.

Immunofluorescence analysis was performed to verify the above results. The results demonstrated that hiPSCs expressed pluripotent markers (NANOG and OCT3/4) on Day 0. On Day 6, the differentiated cells expressed posterior foregut marker CDX2 and epithelial marker EpCAM (Figure 4A). The protein expression levels of ALB and HNF4α were increased from Day 10 to Day 24, which was highly consistent with the corresponding mRNA expression (Figures 4B,C). Interestingly, the protein expression of vimentin (VIM), a mesenchymal marker, emerged sparsely on the surface of the HLOs on Day 10, indicating the existence of non-parenchymal cells. These VIM-positive cell populations increased over the subsequent 2 weeks and ultimately formed an outer shell at the surface of mpHLOs (Figure 4B). Notably, all the cells expressed EpCAM on Day 10, implying their hepatic stem cell identity. Moreover, these EpCAM positive cells decreased over the differentiation and finally existed only in the core of the mpHLOs (Figure 4D). Like the EpCAM expression, CK19 was expressed in nearly all the cells in the HLOs on Day 10 and then expressed only in the core of the HLOs on Day 24. However, these CK19 positive cells experienced the first upregulation and then downregulation in the expression level during the two-week differentiation (Figure 4E). Additionally, AFP didn’t express on Day 10 and then showed on Day 14 in the core of the HLOs (Figure 4D), further confirming the emergence of hepatoblasts. High magnifed images are shown in Supplementary Figure S1.

### 3.4 Functional characterization of micropatterned HLOs

**FIGURE 5:**
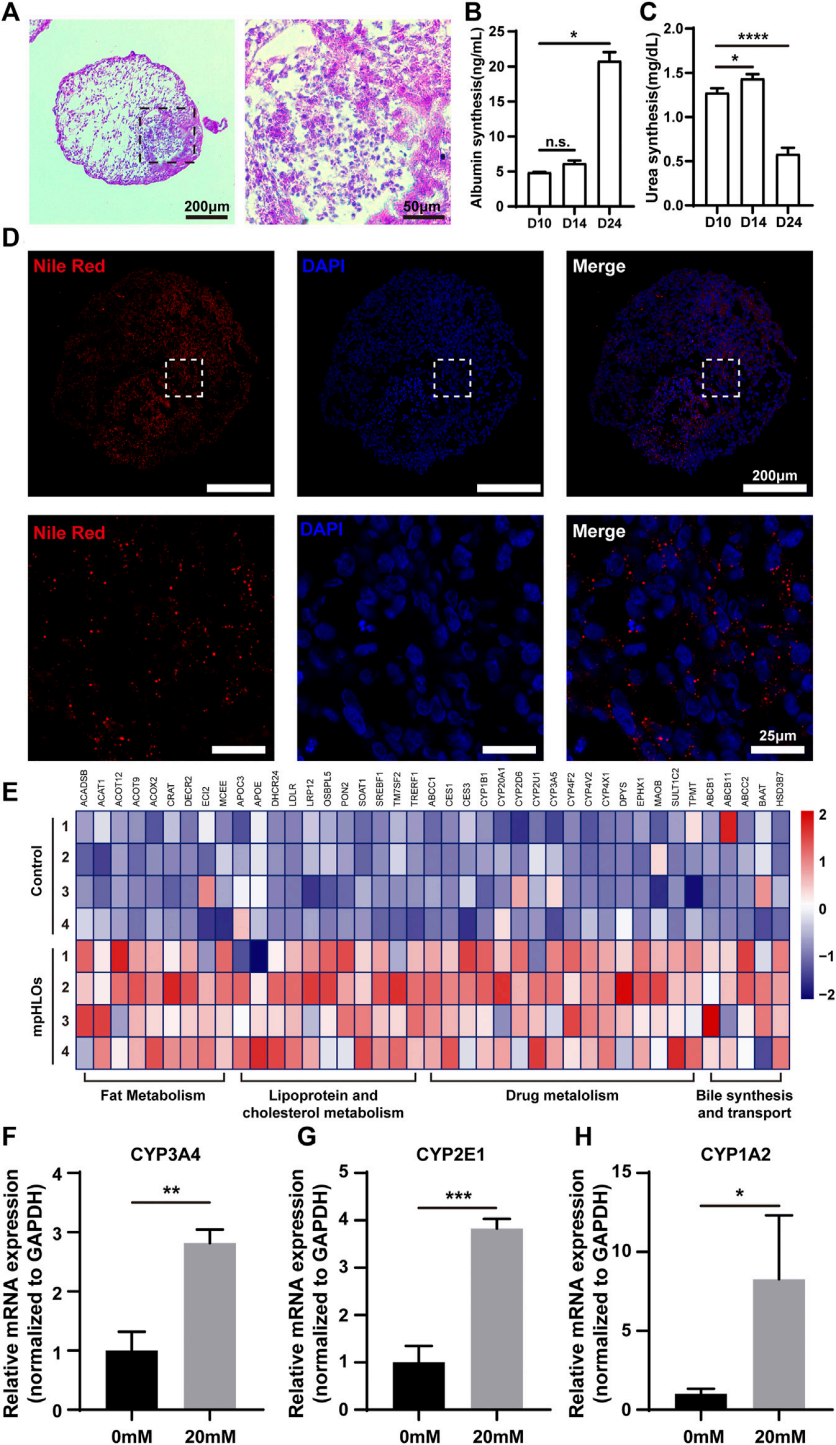
Functional characterization of mpHLOs. **(A)** Glycogen storage of mpHLOs. **(B–C)** Albumin and urea secretion during two-week differentiation. **(D)** Lipid accumulation of mpHLOs. **(E)** Heat map indicates differential gene expression analysis of liver specific function genes. **(F–H)** Relative mRNA expression of CYP450 enzymes (CYP3A4, CYP2E1, and CYP1A2) after 48 h treatment with 0 and 20 mM APAP. Data are shown as the fold changes in expression relative to the levels without treatment. Data are the mean ± SD (*n* = 3) and analyzed by Student’s t test or One-way analysis of variance, *p < 0.05, **p < 0.01, ***p < 0.001, ****p < 0.0001.

We further examined liver-specific functions of the mpHLOs. Specifically, mpHLOs on Day 24 were stained positive by PAS and Nile Red staining assays, indicating their lipid and glycogen synthesis functions (Figures 5A,D). Furthermore, ELISA analysis indicated that albumin in the culture medium increased over the two-week differentiation. Meanwhile, urea was also detected in the supernatant (Figures 5B,C). In addition, the mpHLOs highly expressed genes encoded for proteins and enzymes that are important for essential liver functions, including bile synthesis and transport, lipoprotein and cholesterol metabolism, drug metabolism, and fat metabolism (Figure 5E).

To evaluate the metabolic capacity of mpHLOs, we used the clinical drug APAP to assess the acute toxic effects of a hepatotoxicant on the liver organoids. The liver organoids were treated for 48 h with a 20 mM concentration of APAP, and then RNA was extracted for qPCR. As shown in Figures 5F–H, all three cytochrome P450 enzymes showed higher expression levels after APAP treatment.

## 4 Discussion

PSC-derived HLOs are considered promising humanized organotypic models for mechanistic studies of human liver disease and evaluation of hepatoxicity. However, the heterogeneity of PSC-derived HLOs represents a great challenge that hampers the HLOs from widespread biomedical applications. Here, We address this issue by bioengineering of PSC-derived HLOs with MPCS. Specifically, seeded PSC-derived foregut stem cells can only attach to cell-adhesive microregions determined by the micropatterning technique. When the adhered foregut stem cells reach about 100% cell confluence on the microregions, continuous cell proliferation results in cell stratification and the formation of multilayer cytoarchitecture. After two-week differentiation from Day 10 to Day 24, dome-shaped HLOs are generated with a predefined boundary on the substrate. Additionally, the geometric features such as the interspace and the size of the individual HLOs are determined by the predefined MPCS. We constructed circular mpHLOs with diameters of 100, 200, and 500 μm. Compared to the 500 μm diameter mpHLOs, the 100, and 200 μm mpHLOs displayed same phenotype and protein expression pattern on Day 14 (Supplementary Figure S2), but easily detached from the MPCS during the culture medium exchange. Therefore, we chose to use the 500 μm mpHLOs for subsequent experiments. We design the HLOs with the same size and equal interspace distance from their neighboring HLOs, and the biochemical microenvironments over individual organoids are homogenized. Therefore, the maturity of individual organoids is comparable during the differentation. Moreover, the deterministic locations and controlled sizes of individual organoids facilitate *in-situ* monitoring and whole-mount imaging. The morphology analysis indicates that the area CVs of mpHLOs are significantly lower than that of HLOs in Matrigel domes. Micropatterned substrate is a commonly-used bioengineering approach for tissue culture. Previously, Bhatia’s group employed MPCS to co-culture primary hepatocytes with endothelial cells and fibroblast cells. Cells were self-organized in the patterned microregions and formed a heterocellular liver model, which was applied to emulate the hepatitis viral infection of the adult human liver (March et al., 2015). Additionally, Khetani’s group developed an *in vitro* liver model containing hiPSC-derived hepatic cells and murine embryonic fibroblasts on the micropatterned substrate. They explored this model for long-term drug toxicity assessment (Ware et al., 2015). Compared to these co-culture systems, We differentiated FSCs *in situ* into mpHLO spontaneously containing liver parenchymal and non-parenchymal cells without addition of non-isogenic cells. Moreover, the formation of mpHLOs displays a similar differentiation process to liver development *in vivo*.

Our liver organoids exhibit a strong hepatic fate, but they are immature in functionalities and demonstrate fetal liver features. Immunofluorescence analysis indicates that the mpHLOs are stained intensely for CK19 and EpCAM, weakly for ALB, but negatively for AFP on Day 10 (Figures 4B–E). The protein expression pattern has been previously identified as the phenotypes of hepatic stem cells (hHpSCs) (Schmelzer et al., 2006). hHpSCs are located in the canals of Hering, which is a stem niche in liver (Roskams et al., 2004; Schmelzer et al., 2007). On Day 14, most of the cells in the HLOs positively expressed albumin, AFP, and CK19, which is similar to the expression pattern of hepatoblast in the fetal liver. On Day 24, mpHLOs were stained strongly for ALB, HNF4α, weakly for EpCAM, AFP, and CK19. Hepatoblasts are the dominant cell population in fetal and neonatal livers, and they decline in numbers with age and are found as < 0.1% in normal adult human liver. Fetal livers express high level of AFP, elevated level of ALB, and low level of CK19. The protein expression pattern of mpHLOs is similar to the expression of fetal and neonatal liver (Schmelzer et al., 2006; Si-Tayeb et al., 2010; Lee et al., 2016). Immunofluorescence staining also demonstrates that non-parenchymal cells are at the periphery of the mpHLOs while hepatoblast and immature hepatocytes are at the interior (Figure 2C). Although not fully matured, mpHLOs display liver-specific functions, including albumin secretion, urea production, glycogen synthesis, and lipid synthesis (Figures 5A–E).

To date, there are only a few models available to study the hepatotoxicity of drugs on human fetuses. Here, our mpHLOs provide an *in vitro* model of the fetal liver for relevant studies. Previous studies demonstrated that Acetaminophen (APAP), the most commonly recommended analgesia in pregnancy, can cross the placenta and cause fetal and maternal hepatocytes damage at toxic doses. Specifically, fetal hepatocytes metabolize APAP into hepatoxic metabolites that cause hepatic necrosis (Wilkes et al., 2005; Laine et al., 2009). In our study, the expression of Cytochrome P450 enzymes was significantly up-regulated after APAP treatment for 48 h (Figure 5F), suggesting that mpHLOs are sensitive to drug treatment and can therefore be used as a model for evaluating drug fetal hepatotoxicity.

We admit that our mpHLOs have some limitations. Compared to primary hepatocyte culture, the maturity of mpHLOs is insufficient. For example, the albumin secretion quantity of mpHLOs is much lower than reported in some previous articles (Wang et al., 2018). In addition, the cytoarchitecture of the mpHLOs is different from the liver-specific functional unit, namely the hepatic lobule. We will address these limitations and improve the fidelity of mpHLOs in their function and cellular structure by employing co-culture or dynamic culture strategies.

## 5 Conclusion

Overall, we develop a novel *in vitro* liver model by bioengineering hiPSC-derived liver organoid. The technique to generate the model is high-throughput and reproducible. The liver organoid is uniform and allows whole-mount imaging under a confocal microscope. We believe that this novel liver organoid model will enable the understanding of drug-induced fetal liver injury and provide an innovative platform for basic research in fetal liver development and diseases.

## Data Availability

All data needed to evaluate the conclusions in the paper are presented in the paper and/or Supplementary Material. More detailed data will be made available to interested investigators upon request. The RNA-seq data accompanying the research has been uploaded to sequence read archive (SRA) with the accession number PRJNA837011.

## References

[B1] Bjornsson E. S. Bergmann O. M. Bjornsson H. K. Kvaran R. B. Olafsson S. (2013). Incidence, presentation, and outcomes in patients with drug-induced liver injury in the general population of Iceland. Gastroenterology 144 (7), 1419–1425.e3. 10.1053/j.gastro.2013.02.006 23419359

[B2] Chalasani N. Bonkovsky H. L. Fontana R. Lee W. Stolz A. Talwalkar J. (2015). Features and outcomes of 899 patients with drug-induced liver injury: The DILIN prospective study. Gastroenterology 148 (7), 1340–1352.e7. 10.1053/j.gastro.2015.03.006 25754159 PMC4446235

[B3] Goulart E. de Caires-Junior L. C. Telles-Silva K. A. Araujo B. H. S. Kobayashi G. S. Musso C. M. (2019). Adult and iPS-derived non-parenchymal cells regulate liver organoid development through differential modulation of Wnt and TGF-β. Stem Cell Res. Ther. 10 (1), 258. 10.1186/s13287-019-1367-x 31416480 PMC6694663

[B4] Hoang P. Wang J. Conklin B. R. Healy K. E. Ma Z. (2018). Generation of spatial-patterned early-developing cardiac organoids using human pluripotent stem cells. Nat. Protoc. 13 (4), 723–737. 10.1038/nprot.2018.006 29543795 PMC6287283

[B5] Kamath P. Kamath A. Ullal S. D. (2021). Liver injury associated with drug intake during pregnancy. World J. Hepatol. 13 (7), 747–762. 10.4254/wjh.v13.i7.747 34367496 PMC8326163

[B6] Laine J. E. Auriola S. Pasanen M. Juvonen R. O. (2009). Acetaminophen bioactivation by human cytochrome P450 enzymes and animal microsomes. Xenobiotica 39 (1), 11–21. 10.1080/00498250802512830 19219744

[B7] Lee D. H. Park J. O. Kim T. S. Kim S. K. Kim T. H. Kim M. C. (2016). LATS-YAP/TAZ controls lineage specification by regulating TGFβ signaling and Hnf4α expression during liver development. Nat. Commun. 7, 11961. 10.1038/ncomms11961 27358050 PMC4931324

[B8] Ma Z. Wang J. Loskill P. Huebsch N. Koo S. Svedlund F. L. (2015). Self-organizing human cardiac microchambers mediated by geometric confinement. Nat. Commun. 6, 7413. 10.1038/ncomms8413 26172574 PMC4503387

[B9] March S. Ramanan V. Trehan K. Ng S. Galstian A. Gural N. (2015). Micropatterned coculture of primary human hepatocytes and supportive cells for the study of hepatotropic pathogens. Nat. Protoc. 10 (12), 2027–2053. 10.1038/nprot.2015.128 26584444 PMC5867906

[B10] Ouchi R. Togo S. Kimura M. Shinozawa T. Koido M. Koike H. (2019). Modeling steatohepatitis in humans with pluripotent stem cell-derived organoids. Cell Metab. 30 (2), 374–384.e6. 10.1016/j.cmet.2019.05.007 31155493 PMC6687537

[B11] Roskams T. A. Theise N. D. Balabaud C. Bhagat G. Bhathal P. S. Bioulac-Sage P. (2004). Nomenclature of the finer branches of the biliary tree: Canals, ductules, and ductular reactions in human livers. Hepatology 39 (6), 1739–1745. 10.1002/hep.20130 15185318

[B12] Schmelzer E. Wauthier E. Reid L. M. (2006). The phenotypes of pluripotent human hepatic progenitors. Stem Cells 24 (8), 1852–1858. 10.1634/stemcells.2006-0036 16627685

[B13] Schmelzer E. Zhang L. Bruce A. Wauthier E. Ludlow J. Yao H. L. (2007). Human hepatic stem cells from fetal and postnatal donors. J. Exp. Med. 204 (8), 1973–1987. 10.1084/jem.20061603 17664288 PMC2118675

[B14] Sgro C. Clinard F. Ouazir K. Chanay H. Allard C. Guilleminet C. (2002). Incidence of drug-induced hepatic injuries: A French population-based study. Hepatology 36 (2), 451–455. 10.1053/jhep.2002.34857 12143055

[B15] Shen T. Liu Y. Shang J. Xie Q. Li J. Yan M. (2019). Incidence and etiology of drug-induced liver injury in mainland China. Gastroenterology 156 (8), 2230–2241.e11. 10.1053/j.gastro.2019.02.002 30742832

[B16] Shinozawa T. Kimura M. Cai Y. Saiki N. Yoneyama Y. Ouchi R. (2021). High-fidelity drug-induced liver injury screen using human pluripotent stem cell-derived organoids. Gastroenterology 160 (3), 831–846.e10. 10.1053/j.gastro.2020.10.002 33039464 PMC7878295

[B17] Si-Tayeb K. Lemaigre F. P. Duncan S. A. (2010). Organogenesis and development of the liver. Dev. Cell 18 (2), 175–189. 10.1016/j.devcel.2010.01.011 20159590

[B18] Thompson W. L. Takebe T. (2020). Generation of multi-cellular human liver organoids from pluripotent stem cells. Methods Cell Biol. 159, 47–68. 10.1016/bs.mcb.2020.03.009 32586449 PMC8011941

[B19] Velazquez J. J. LeGraw R. Moghadam F. Tan Y. Kilbourne J. Maggiore J. C. (2021). Gene regulatory network analysis and engineering directs development and vascularization of multilineage human liver organoids. Cell Syst. 12 (1), 41–55.e11. 10.1016/j.cels.2020.11.002 33290741 PMC8164844

[B20] Wang Y. Wang H. Deng P. Chen W. Guo Y. Tao T. (2018). *In situ* differentiation and generation of functional liver organoids from human iPSCs in a 3D perfusable chip system. Lab. Chip 18 (23), 3606–3616. 10.1039/c8lc00869h 30357207

[B21] Ware B. R. Berger D. R. Khetani S. R. (2015). Prediction of drug-induced liver injury in micropatterned Co-cultures containing iPSC-derived human hepatocytes. Toxicol. Sci. 145 (2), 252–262. 10.1093/toxsci/kfv048 25716675

[B22] Wilkes J. M. Clark L. E. Herrera J. L. (2005). Acetaminophen overdose in pregnancy. South. Med. J. 98 (11), 1118–1122. 10.1097/01.smj.0000184792.15407.51 16351032

[B23] Xu J. Pan D. Liao W. Jia Z. Pan M. Weng J. (2022). Application of 3D hepatic plate-like liver model for statin-induced hepatotoxicity evaluation. Front. Bioeng. Biotechnol. 10, 826093. 10.3389/fbioe.2022.826093 35372314 PMC8968918

