# Supplementary material for: High-throughput bioengineering of homogenous and functional human-induced pluripotent stem cells-derived liver organoids via micropatterning technique
Source: Front Bioeng Biotechnol. 2022 Aug 10;10:937595. doi: 10.3389/fbioe.2022.937595 (PMC9399390; doi:10.3389/fbioe.2022.937595)
Supplement: Supplementary file 1 [file DataSheet1.docx]

Supplementary Material


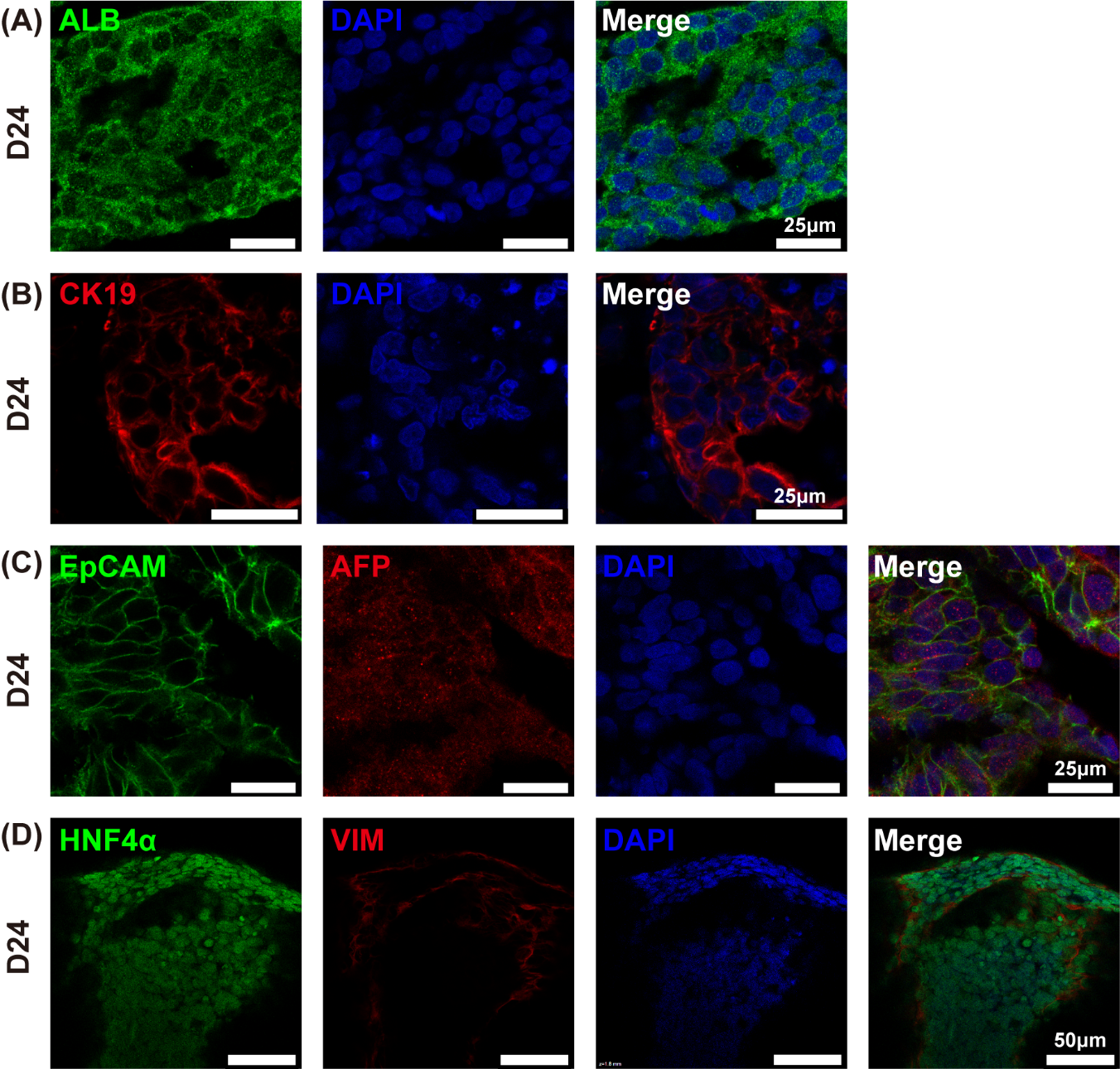


Figure S1. High magnifed images of immunofluorescence staining of mpHLOs for (A) ALB, (B) CK19, (C) EpCAM and AFP, and (D) HNF4α and VIM.


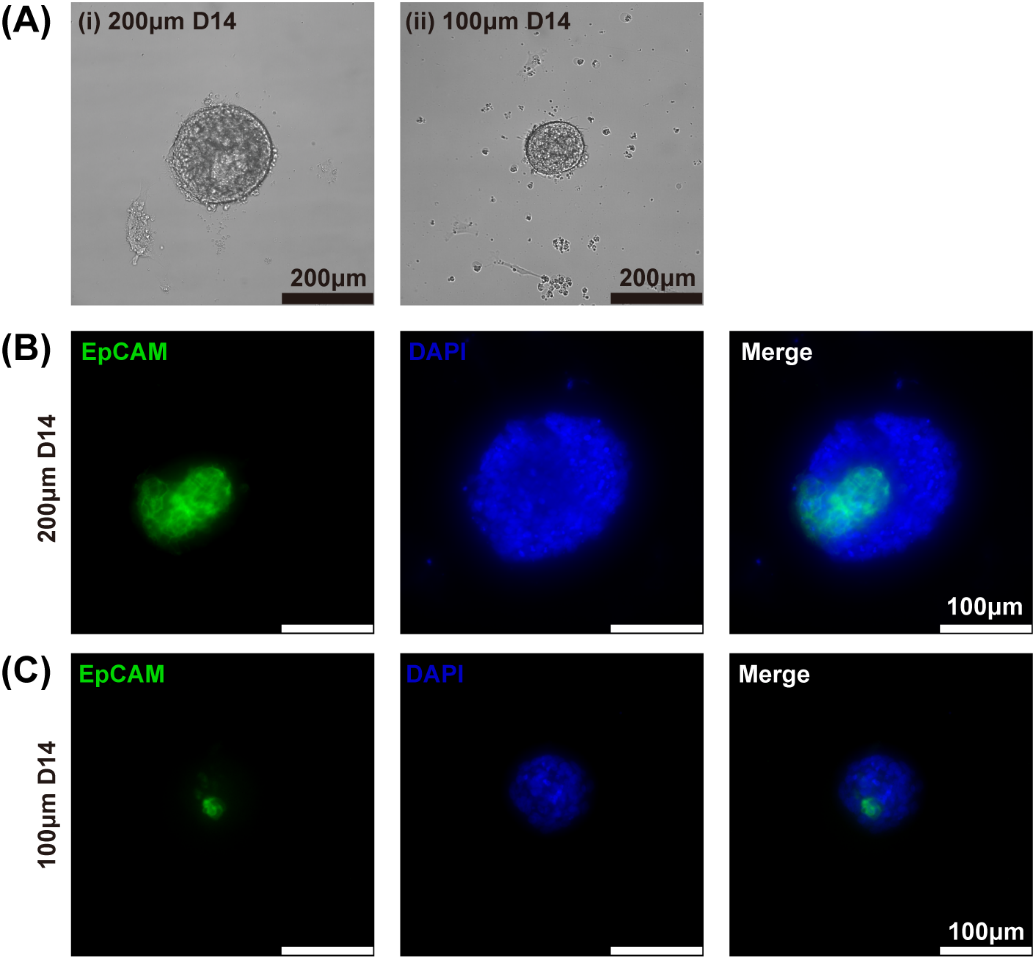


Figure S2. Circular mpHLOs with 200 μm and 100 μm diameters. (A) Bright-field images of circular mpHLOs on Day 14. (B) Immunofluorescence staining of circular mpHLOs for EpCAM.

Table S1. Key resources

| Media Name | Component | | Concentration | | Source | | | Identifier |
| --- | --- | --- | --- | --- | --- | --- | --- | --- |
| Day 1 Medium | RPMI 1640 medium | | 500 mL | | Gibco | | | 11875 |
|  | Pen/Strep | | 1% (v/v) | | Gibco | | | 15140-122 |
|  | Hepes | | 25 mM | | Gibco | | | 15630106 |
|  | BMP4 | | 50 ng/mL | | R&D Systems | | | 314-BP-050 |
|  | Activin A | | 100 ng/mL | | CELL guidance systems | | | GFH6-10*10 |
| Day 2 Medium | RPMI 1640 medium | | 500 mL | | Gibco | | | 11875 |
|  | Pen/Strep | | 1% (v/v) | | Gibco | | | 15140-122 |
|  | Hepes | | 25 mM | | Gibco | | | 15630106 |
|  | Activin A | | 100 ng/mL | | CELL guidance systems | | | GFH6-10*10 |
|  | KnockOut™ Serum Replacement | | 0.2% (v/v) | | Gibco | | | A3181501 |
| Day 3 Medium | RPMI 1640 medium | | 500 mL | | Gibco | | | 11875 |
|  | Pen/Strep | | 1% (v/v) | | Gibco | | | 15140-122 |
|  | Hepes | | 25 mM | | Gibco | | | 15630106 |
|  | Activin A | | 100 ng/mL | | CELL guidance systems | | | GFH6-10*10 |
|  | KnockOut™ Serum Replacement | | 2% (v/v) | | Gibco | | | A3181501 |
| Day 4 to 6 Medium | Advanced DMEM/F12 | | 500 mL | | Thermo Fisher | | | 12634010 |
|  | B-27^TM^ Supplement | | 1% (v/v) | | Thermo Fisher | | | 17504-044 |
|  | N2 | | 1% (v/v) | | Thermo Fisher | | | 17502-048 |
|  | Hepes | | 10 mM | | Gibco | | | 15630106 |
|  | Glutamax | | 1% (v/v) | | Thermo Fisher | | | 35050 |
|  | Pen/Strep | | 1% (v/v) | | Gibco | | | 15140-122 |
|  | FGF2 | | 500 ng/mL | | Source Bioscience | | | LS-G16657-2 |
|  | CHIR99021 | | 3 μM | | Stemgent | | | 04-0004-10 |
| Liver Organoid Formation Medium | Advanced DMEM/F12 | | 500 mL | | Thermo Fisher | | | 12634010 |
|  | B-27^TM^ Supplement | | 1% (v/v) | | Thermo Fisher | | | 17504-044 |
|  | N2 | | 1% (v/v) | | Thermo Fisher | | | 17502-048 |
|  | Hepes | | 10 mM | | Gibco | | | 15630106 |
|  | Glutamax | | 1% (v/v) | | Thermo Fisher | | | 35050 |
|  | Pen/Strep | | 1% (v/v) | | Gibco | | | 15140-122 |
|  | FGF2 | | 80 ng/mL | | Source Bioscience | | | LS-G16657-2 |
|  | CHIR99021 | | 3 μM | | Stemgent | | | 04-0004-10 |
| Liver Organoid Specification Medium | Advanced DMEM/F12 | | 500 mL | | Thermo Fisher | | | 12634010 |
|  | B-27^TM^ Supplement | | 1% (v/v) | | Thermo Fisher | | | 17504-044 |
|  | N2 | | 1% (v/v) | | Thermo Fisher | | | 17502-048 |
|  | Hepes | | 10 mM | | Gibco | | | 15630106 |
|  | Glutamax | | 1% (v/v) | | Thermo Fisher | | | 35050 |
|  | Pen/Strep | | 1% (v/v) | | Gibco | | | 15140-122 |
|  | RA | | 2 μM | | Sigma-Aldrich | | | R2625-50MG |
| Complete Hepatocyte Culture Medium | Hepatocyte Culture Medium | | 500 mL | | Lonza | | | CC-3199 |
|  | HGF | | 10 ng/mL | | Peprotech | | | 100-39 |
|  | Dexamethasone | | 100 nM | | Sigma-Aldrich | | | D4902-25MG |
|  | OSM | | 20 ng/mL | | R&D Systems | | | 8475-OM-050 |
| Antibodies | | **Dilution ratio** | **Source** | | | | **Identifier** | |
| NANOG | | 1:20 | R&D Systems | | | | AF1997 | |
| OCT3/4 | | 1:50 | Santa Cruz Biotechnology | | | | sc-5279 | |
| CDX2 | | 1:200 | Abclonal | | | | A19030 | |
| EpCAM | | 1:800 | Cell Signaling Technology | | | | 2929S | |
| AFP | | 1:1000 | Dako | | | | A008 | |
| CK19 | | 1:200 | Abcam | | | | ab76539 | |
| ALB | | 1:200 | Bethy | | | | A80-129A | |
| HNF4α | | 1:250 | Santa Cruz | | | | sc-374229 | |
| VIM | | 1:200 | ABclonal Technology | | | | A19607 | |
| Alexa Fluor 488 Donkey anti-Goat | | 1:200 | Invitrogen | | | | A11055 | |
| Alexa Fluor 488 Donkey anti-Mouse | | 1:200 | Invitrogen | | | | A21202 | |
| Alexa Fluor 568 Donkey anti-Rabbit | | 1:200 | Invitrogen | | | | A10042 | |
| Alexa Fluor 568 Donkey anti-Mouse | | 1:200 | Invitrogen | | | | A10037 | |
| Gene | | **Forward Primer** | | | | **Reverse Primer** | | |
| GAPDH | | GAAGGTGAAGGTCGGAGTC | | | | GAAGATGGTGATGGGATTTC | | |
| NANOG | | CCTGAAGACGTGTGAAGATGAG | | | | GCTGATTAGGCTCCAACCATA | | |
| OCT4 | | CAAAGCAGAAACCCTCGTGC | | | | TCTCACTCGGTTCTCGATACTG | | |
| CDX2 | | TGGAGCTGGAGAAGGAGTTT | | | | CTGCTGCTGCTGTTGCTG | | |
| AFP | | ACAAAAAGCCCACTCCAGCA | | | | ATGGCTTGGAAAGTTCGGGT | | |
| CK19 | | CGCGGCGTATCCGTGTCCTC | | | | AGCCTGTTCCGTCTCAAACTT | | |
| ALB | | TGCTGAGGCAAAGGATGTCT | | | | ATGTCTTGGCAAGTCTCAGCA | | |
| HNF4α | | TGCAGGTGTTGACGATGGGCA | | | | ACCACGCACTGCCGGCTAAAT | | |
| CYP3A4 | | GGTGGTGAATGAAACGCTCAG | | | | CACCCCTTTGGGAATGAACA | | |
| CYP2E1 | | CCAGCTTTCTACACTACTTGCC | | | | GGTCCAGAGATTGATGGTGCT | | |
| CYP1A2 | | CTTCGCTACCTGCCTAACCC | | | | GACTGTGTCAAATCCTGCTCC | | |
| Reagent or Kit | | **Source** | | **Identifier** | | | | |
| SU-8 photoresist | | MicroChem | | SU-8 2015 | | | | |
| PDMS prepolymer | | Momentive | | RTV615 | | | | |
| PEG 1000 | | Polysciences | | 16666 | | | | |
| PEG 400 | | Polysciences | | 01871 | | | | |
| Isopropanol | | HUSHI | | 80109218 | | | | |
| Irgacure 2959 | | Sigma | | 410896-10G | | | | |
| Nuwacell^TM^ ncTarget hPSC Medium | | Nuwacell Biotechnologies | | RP01020 | | | | |
| Accutase | | Gibco | | A1110501 | | | | |
| Vitronectin | | Gibco | | A31804 | | | | |
| Y-27632 | | STEMCELL Technologies | | 72304 | | | | |
| 10×DPBS | | Gibco | | 14200166 | | | | |
| Matrigel | | Corning | | 356237 | | | | |
| Paraformaldehyde | | Sigma-Aldrich | | P6148 | | | | |
| Bovine Serum Albumin | | Sigma Life Science | | B2064-50G | | | | |
| 4,6-diamidino-2-phenylindole | | Thermo Fisher | | P36931 | | | | |
| Triton X-100 | | HUSHI | | 30188928 | | | | |
| Total RNA Extraction Reagent (Trizol) | | Abclonal | | RK30129 | | | | |
| ABScript Ⅲ RT Master Mix for qPCR | | ABclonal | | RK20428 | | | | |
| 2X Universal SYBR Green Fast qPCR Mix | | ABclonal | | RM21203 | | | | |
| Human Albumin ELISA Kit | | Bethyl Laboratories | | E80-129 | | | | |
| QuantiChrom^TM^ Urea Assay Kit | | Bioassay Systems | | DIUR-500 | | | | |
| Sucrose | | HUSHI | | 10021418 | | | | |
| Periodic-acid-Schiff reagent | | Leagene | | DG0011 | | | | |
| Nile Red | | Sigma-Aldrich | | 19123 | | | | |
| Equipment | | **Source** | | **Identifier** | | | | |
| Photolithography machine | | Shanghai Xueze Optical Machinery | | JKG-2A | | | | |
| Plasma cleaner | | MING HENG | | PDC-MG | | | | |
| Confocal microscope | | Leica | | TCS SP8 STED | | | | |
| Inverted biological microscope | | Sunny Optical Technology | | ICX41 | | | | |
| Epi-fluorescence microscope | | Olympus | | IX-83 | | | | |
| Cryostat | | Leica | | CM1900 | | | | |
